# Role of Portosystemic Shunt and Portal Vein Stent in Managing Portal Hypertension Due to Hematological Diseases

**DOI:** 10.7759/cureus.54206

**Published:** 2024-02-14

**Authors:** Ji Hoon Kim, Suho Kim, Hee-Chul Nam, Chang Wook Kim, Jae-Sung Yoo, Ji Won Han, Jeong Won Jang, Jong Young Choi, Seung Kew Yoon, Ho-Jong Chun, Sung-Eun Lee, Jung-Suk Oh, Pil Soo Sung

**Affiliations:** 1 Gastroenterology and Hepatology, Uijeongbu St. Mary's Hospital, Uijeongbu, KOR; 2 Radiology, Seoul St. Mary's Hospital, Seoul, KOR; 3 The Catholic University of Korea, Internal Medicine, Uijeongbu, KOR; 4 Gastroenterology and Hepatology, Seoul St. Mary's Hospital, Seoul, KOR; 5 Gastroenterology and Hepatology, Seoul St. mary's Hospital, Seoul, KOR; 6 Hematology, Seoul St. Mary's Hospital, Seoul, KOR

**Keywords:** transjugular intrahepatic portosystemic shunt (tips), portal vein stenting, venous varix, hematological malignancies, myeloproliferative neoplasm disease

## Abstract

Introduction: Patients with hematological diseases experience complications related to portal hypertension, including life-threatening complications such as variceal bleeding.

Methods: We analyzed the prognosis of patients with hematological diseases and portal hypertension treated with transjugular intrahepatic portosystemic shunts (TIPS) or portal vein stents. We retrospectively assessed patients with hematological diseases and portal hypertension who had variceal bleeding. We evaluated the characteristics and prognosis of the enrolled patients. A total of 11 patients with hematological diseases who underwent TIPS, or portal vein stenting, were evaluated.

Results: The median follow-up period was 420 days. Of the 11 patients, eight showed resolution of portal hypertension and its complications following TIPS, or stent insertion. One patient experienced rebleeding due to incomplete resolution of portal hypertension, and two other patients also experienced rebleeding because they underwent TIPS closure or revision due to repetitive hepatic encephalopathy.

Conclusion: Portosystemic shunt and stent installation are effective treatment options for portal hypertension due to hematological diseases.

## Introduction

Portal hypertension is the increase in pressure of the portal venous system; it is often defined as the state where the pressure gradient between the portal vein and the inferior vena cava is greater than 5 mmHg, indicating sinusoidal portal hypertension [1]. The main cause of portal hypertension is liver cirrhosis, with other causes being less common (<10%) [2]. Causes of portal hypertension are often classified into three categories: prehepatic, intrahepatic, and posthepatic. Prehepatic portal hypertension includes portal hypertension due to portal vein thrombosis caused by infections or prothrombotic diseases, including congenital diseases such as protein C or S deficiency, or due to the hypercoagulable status of hematological diseases [3]. In addition, intrahepatic obstructions due to extramedullary myeloid metaplasia and sinusoidal changes secondary to hematological diseases may also lead to portal hypertension. Although ideally, the hepatic venous pressure gradient is the established method to accurately measure the portal pressure, the invasive nature of such tests does not allow for regular testing, and many studies are being conducted regarding this conundrum [4]. Among patients diagnosed with myeloproliferative neoplasms (MPNs), 7% to 18% suffer from portal hypertension and frequently present with variceal bleeding or refractory ascites [5]. In addition to MPNs, there has been a case report of extramedullary hematopoiesis in the liver tissue leading to portal hypertension in patients diagnosed with myelodysplastic syndrome (MDS) [6]. It is worth noting that there is no established treatment or intervention for treating patients with hematological diseases suffering from complications due to portal hypertension. On the other hand, it has been well established that a transjugular intrahepatic portosystemic shunt (TIPS) is helpful in relieving portal hypertension and can be used in refractory ascites and recurrent variceal bleeding [7]. A recent study by Larrue et al. provided evidence that TIPS is effective in reducing the rate of refractory ascites and recurrent bleeding in patients with portal hypertension and cirrhosis [8].

Few studies have investigated the relationship between hematological diseases and portal hypertension [9,10]. In 2020, Rössle et al. reported interventional treatments, including portal vein stents, as an effective method for managing portal vein thrombosis in non-cirrhotic patients [11]. In 2022, Monsour et al. reported that invasive recanalization, including TIPS, is an effective method for managing patients with portal vein thrombosis [12]. As an institute with one of the world's largest hematology centers, we conducted a retrospective study on the resolution of portal hypertension in patients diagnosed with hematological diseases.

## Materials and methods

This is a retrospective, single-center study. It was approved by the Institutional Review Board of Seoul St. Mary’s Hospital (approval no. KC23RISI0654). The study conformed to the ethical guidelines of the Helsinki Declaration.

We identified 4,276 patients diagnosed with hematological diseases between January 2012 and January 2022. Patients without radiological or endoscopic findings suggestive of portal hypertension were excluded. Radiological findings suggestive of portal hypertension included esophageal varices, gastric varicies, and ascites, and endoscopic findings suggestive of portal hypertension included endoscopic lesions, as well as esophageal varices and gastric varices. Patients with a history of hepatitis B virus (HBV) infection, hepatitis C virus (HCV) infection, or chronic alcohol consumption were excluded. All patients with radiological findings and endoscopic findings received radiological interventions, including TIPS or portal vein stent insertion, due to recurrent esophageal varix bleeding. Finally, 11 patients were enrolled in the study. Their complete blood counts, blood chemistry, endoscopy reports, and CT scans were analyzed.

TIPS procedure protocol

After localization of the portal vein, the right hepatic vein is catheterized transjugularly via a 0.018-inch hair guidewire. An 11F introducer sheath is inserted over the wire, and the hepatic vein is cannulated. The portal vein near the bifurcation site is punctured with a Colapinto needle, and a 5F Kumpe (KMP) catheter is then inserted into the portal vein via the hepatic-parenchymal-portal vein pathway, and the parenchymal tract is dilated with an 8cc-4cm balloon catheter (Armada™, Abbott, Chicago, IL, USA). Via fluoroscopic guidance, after the assurance of puncture of the portal vein, a catheter is introduced via guidewire, and the portal venous pressure gradient is calculated. Then, the introducer sheath is inserted, after which the 10 mm-8 cm self-expandable stent, graft-stent (SEAL stent-graft, S&G Biotech., Seongnam, Korea), is deployed and expanded. Post-stent dilatation is performed with the 8 mm-4 cm balloon catheter (Armada). Then, the shunt is installed.

Portal vein stent protocol

After the puncture of the splenic vein, a hair guide wire is introduced. A microsheath is inserted, and a 0.035-inch guidewire is then introduced, followed by a 5F catheter. After a splenic venogram, a microcatheter is maneuvered at the location of the portal vein complication site. Via ballooning, the portal vein is pre-dilated, following bare metal stent insertion.

Follow up

After the TIPS procedure, or portal vein stent insertion, the patients were followed up regularly via the outpatient department with blood tests and imaging, including a complete blood count, blood chemistry, endoscopy, and abdomen sonography with color Doppler. All statistical analyses were performed using SPSS Statistics version 23.0 (IBM Corp., Armonk, NY, USA).

## Results

Baseline characteristics

The baseline characteristics of the enrolled patients are summarized in Table 1. Among the enrolled patients, two were male. The mean age of the enrolled patients was 53.64±9.82 years. Three patients were diagnosed with primary myelofibrosis (PMF), all of whom harbored the JAK2 V617F mutation. Four patients were diagnosed with MDS, two with multiple myeloma (MM), one with essential thrombocythemia (ET), and one with polycythemia vera (PV). None of the patients had hepatitis B virus (HBV) or hepatitis C virus (HCV) infections, which may have caused liver parenchymal disease. The CT scans and US of all the enrolled patients revealed no sign of fatty liver; the median BMI was 20.28 and the mean BMI was 22.06. The median spleen size was 11.5 cm, and the mean spleen size was 14.73 cm (one patient received a splenectomy). Four patients had portal vein thrombosis; another four patients had a history of treatment for their hematological disease; two patients were treated with eculizumab; and one patient was treated with ruxolitinib. One patient had a history of bone marrow transplantation (BMT). Seven patients had received no previous treatment for hematological diseases.

**Table 1:** Baseline characteristics of the enrolled patients Data are presented as n (%) and means ± standard deviations MDS: Myelodysplastic syndrome; MM: Multiple myeloma; ET: Essential thrombocythemia; HBsAg, Hepatitis B surface antigen; Anti-HCV Ab: Anti-hepatitis C virus antibody; DM: Diabetes mellitus; BMT: Bone marrow transplant

Characteristics	N (%) (Total n = 11)
Sex (M/F)^†^	2( 18%)/9 (82%)
Age	53.64±9.82
Etiology	
Primary myelofibrosis	3 (27.27%)
JAK2 V617F mutation	3 (27.27%)
MDS	4 (36.36%)
MM	2 (18.18%)
ET	1 (9.09%)
Polycythemia vera	1 (9.09%)
HBsAg^‡^	0
Anti-HCV Ab^§^	0
History of DM	3 (27.27%)
BMI	22.06±3.83
Portal vein thrombosis	4 (36.36%)
Spleen size	14.73±7.33
Treatment	
Eculizumab	2 (18.18%)
Ruxolitinib	1 (9.09%)
History of BMT^¶^	1 (9.09%)
No history of chemotherapy or BMT	7 (63.63%)

Changes in liver functions and prognosis after TIPS or portal vein stent

The changes in liver function and prognosis of the enrolled patients are also shown in Table 2. Six patients were treated with TIPS, and five were treated with portal vein stent insertion. Out of the five patients treated with portal vein stent insertion, four patients were treated with portal vein stents because of portal vein thrombosis, and one patient had idiopathic portal vein occlusion. The mean hepatic venous pressure gradient was 16.33 mmHg before the TIPS procedure and 6.67 mmHg after the procedure. The initial laboratory tests (before TIPS or portal vein stent insertion) revealed a median total bilirubin of 0.88 mg/dL, a median albumin of 3.7 g/dL, and a median international normalized ratio (INR) of 1.17. The laboratory tests at the six-month follow-up period after TIPS or portal vein stent insertion showed a median bilirubin of 0.63 mg/dL, a median albumin of 3.6 g/dL, and a median INR of 1.14. No significant clinical changes concerning laboratory tests were observed. Of the 11 enrolled patients, five died, although none of the patients died of portal hypertension-related complications. Four patients expired due to septic shock; one patient expired due to intracranial hemorrhage. The mean time to death was 796 days, and the median time to death was 420 days.

**Table 2:** Prognosis and changes in liver function of the enrolled patients Data are presented as n (%) and means ± standard deviations TIPS: Transjugular intrahepatic portosystemic shunt; INR: International normalized ratio; PTH: Portal hypertension

Parameters	Values
Treatment of portal hypertension	
TIPS^†^	6
Portal vein stent	5
Pre-TIPS pressure gradient (mmHg)	16.33±5.43
Post-TIPS pressure gradient (mmHg)	6.67±4.59
Initial lab	
Median total bilirubin (mg/dL)	0.88
Median albumin (g/dL)	3.7
Median INR^‡^	1.17
Last lab	
Median total bilirubin (mg/dL)	0.63
Median albumin (g/dL)	3.6
Median INR	1.14
Deaths	5
Time to death	796±827.53
Portal hypertension-related mortality	0
Intracranial hemorrhage	1
Septic shock	4
Rebleeding	3
Unresolved PTH after TIPS	1
Varix bleeding after TIPS closure	2
Hepatic encephalopathy	2
Patency of TIPS or portal vein stent	
Patent	8
Failure of the procedure of closure	3

Three patients experienced rebleeds. One patient showed an incomplete resolution of the high portal pressure after TIPS; the pressure gradient decreased to only 15 mmHg from 17 mmHg after the procedure. The other two patients who experienced recurrent varix bleeding had received TIPS closure due to complications related to the TIPS procedure. Both suffered from hepatic encephalopathy, apparent from elevated ammonia levels and physical examination, including flapping tremors. These patients were treated with esophageal varices ligation. The hematological parameters of the enrolled patients are described in Table 3.

**Table 3:** Complete blood counts and blast percentages of enrolled patients

Parameters	Values
Hemoglobin (g/dL)	9.8
Hematocrit (%)	31.5
Platelet (/uL)	107000
Blast (%)	1%
White blood cell counts (/uL)	5460

Exemplary cases

Case 1

**Figure 1:**
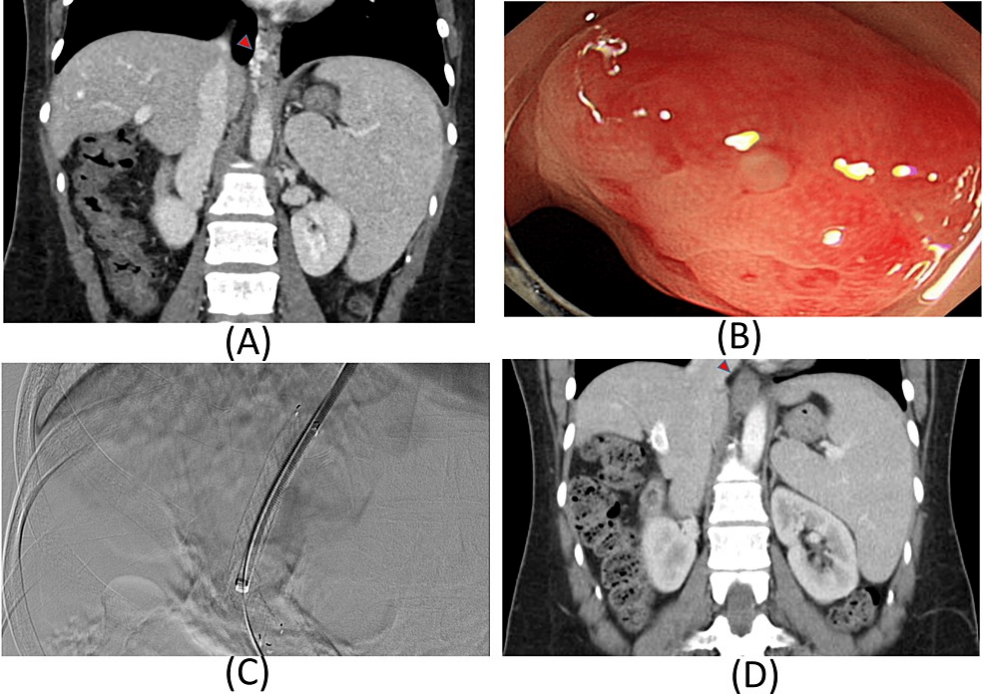
CT scans and intraoperative view of the TIPS procedure A: Coronal view of the CT scan showing splenomegaly and varices but no sign of cirrhosis; B: Esophagogastroduodenoscopy showing esophageal varix with 'white nipple sign' at the gastroesophageal junction; C: TIPS procedure; D: Coronal view of the CT scan showing resolution of previously noted varices with patent TIPS stent MDS: Myelodysplastic syndrome; TIPS: Transjugular intrahepatic portosystemic shunt

A 34-year-old female patient diagnosed with MDS visited the ED, presenting with recurrent melena and dizziness. The CT revealed no evidence of liver cirrhosis, but esophageal varices could be observed (Figure 1A). Esophagogastroduodenoscopy revealed esophageal varices, and a recent bleeding stigmata was observed; an esophageal varix ligation was performed (Figure 1B). Considering the high chance that the portal hypertension was due to extramedullary myeloid metaplasia leading to recurrent bleeding, the installation of a TIPS was decided. During the procedure, the pressure gradient between the right hepatic vein (RHV) and main portal vein (MPV) was reduced from 17 mmHg to 7 mmHg (Figure 1C). Until the last follow-up, the patient had not experienced recurrent bleeding or encephalopathy, and a follow-up color Doppler US showed a patent and well-flowing shunt. Follow-up CT scans showed no evidence of previously observed varices (Figure 1D).

Case 2

**Figure 2:**
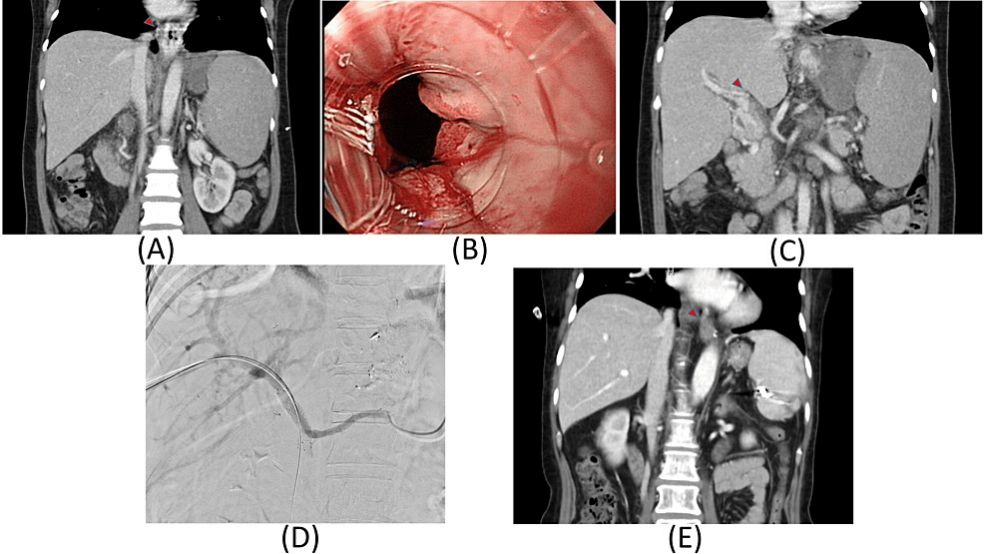
CT scans and intraoperative view of the portal vein stent insertion procedure A: Coronal view of the CT scan showing splenomegaly and varices but no sign of cirrhosis; B: Esophagogastroduodenoscopy showing esophageal varix with 'white nipple sign' at the gastroesophageal junction; C: Coronal view of the CT scan showing portal vein thrombosis; D: Portal vein stent insertion procedure; E: Coronal view of the CT scan showing resolution of previously noted varices

A 50-year-old female patient diagnosed with polycythemia vera visited the ED, presenting with hematemesis and melena. The CT revealed no sign of liver cirrhosis but showed signs of esophageal varices (Figure 2A). Esophagogastroduodenoscopy revealed esophageal varices, a recent bleeding stigmata was observed, and esophageal varix ligation was performed (Figure 2B). The CT scan also showed portal vein thrombosis, which was thought to be the cause of portal hypertension leading to esophageal varix formation (Figure 2C). The patient was discharged after stabilization. After two more varix bleeding events, a portal vein stent was installed (Figure 2D). Until the last follow-up, the patient presented no signs of additional bleeding, and the follow-up CT scan revealed the resolution of esophageal varices (Figure 2E).

## Discussion

A significant proportion of patients with hematological diseases experience portal hypertension caused by diffuse myeloid metaplasia and the prothrombotic nature of hematological diseases [5]. Normally, the increased resistance of blood flow to the portal vein occurs via two mechanisms: the structural component and the dynamic component. Distortion of the hepatic circulation may occur due to fibrosis, vascular occlusion, or myeloid metaplasia, leading to structural resistance [3].

In fact, extramedullary myeloid metaplasia is possible in organs such as the liver, spleen, and lymph nodes in MPNs [13] . Extramedullary myeloid metaplasia leads to portal hypertension, and similar to cirrhosis, the first clinical sign is often varix bleeding [14]. Additionally, changes in blood dynamics occur due to the contractures of the blood vessels as a result of structural changes [3]. Because of these changes, an increase in splanchnic blood flow occurs, and there is an increased release of vascular endothelial growth factor (VEGF), nitric oxide, and other splanchnic vasodilators, resulting in the worsening of portal hypertension [3]. In patients with hematological diseases, portal hypertension may occur due to diffuse myeloid metaplasia or thrombosis. In 2012, Barbui et al. reported the JAK2 V617F mutation as a significant risk factor for thrombosis in MPN patients [15]. Moreover, multiple studies conducted by Campbell et al. and Xavier et al. have suggested significantly increased venous thrombosis in JAK2 V617F-positive patients [16,17]. However, few studies have been conducted on the management of portal hypertension due to hematological diseases, and our institution recognized the need to investigate this conundrum. In 2017, Reilly et al. conducted a multicenter study suggesting that TIPS should be considered an effective management method for complications arising from portal hypertension due to MPN [18]. Our study included portal vein stents as well as TIPS, whereas Reilly et al. only included the TIPS procedure. In 2020, Lee et al. also reported TIPS to be effective in treating complications due to portal hypertension from MPN [10]. In 2022, Aspite et al. reported three cases in which portal hypertension complications in patients diagnosed with myelofibrosis with hepatic myeloid metaplasia were resolved using TIPS [19]. Whereas Aspite et al. only involved TIPS procedures, our study included portal vein stents as well as TIPS.

Our study included patients with various hematological diseases, including PMF (all of whom had JAK2 V617F mutations), MDS, MM, ET, and PV. Four patients had portal vein thrombosis, and portal hypertension was assumed to have resulted from the thrombosis, although diffuse myeloid metaplasia could have also been a cause. The remaining six patients did not have portal vein thrombosis, implying that their portal hypertension was solely due to diffuse myeloid metaplasia. Six patients underwent TIPS, and five patients underwent portal vein stent installation. Five patients died by the end of the follow-up period; however, none of the deaths were associated with complications due to portal hypertension. Two of our patients were receiving eculizumab and ruxolitinib, both of which are known to decrease the prevalence of thrombosis. Accordingly, these patients did not develop thrombosis. In our study, all but one patient experienced resolution of complications due to portal hypertension, suggesting radiological interventions, including TIPS and portal vein stent installation, may be warranted in the management of complications due to portal hypertension in patients with hematological diseases.

The TIPS procedure relieves portal hypertension by installing a shunt between the portal vein and the hepatic vein. A portal vein stent is the installation of a stent in the portal vein when an occlusion is observed. Both procedures help alleviate portal hypertension and therefore increase the central blood volume [20]. Physicians should be aware of the hemodynamic nature of these invasive procedures, especially TIPS. The TIPS installation leads to an increase in preload and has the potential to impair cardiac function [21].

This study had some limitations. First, it is retrospective in nature. Second, considering that there are no definite guidelines regarding complications due to portal hypertension in non-cirrhotic patients, invasive procedures, such as TIPS and portal vein stent insertion, were not performed for non-fatal complications. To our knowledge, there have been no cases of recurrent ascites due to portal hypertension resulting from hematological diseases at our institution. Ascites in patients with hematological diseases is very rare and is often a complication of peritoneal myeloid metaplasia and not portal hypertension [22,23]. Third, it is worth mentioning that splenomegaly may also contribute to the aggravation of portal hypertension [24]. However, considering the improvement of our enrolled patients, it is logical to assume that TIPS and portal vein stent placement are enough to counter its effect. However, more studies are required in this area.

## Conclusions

Currently, there is no guideline for managing portal hypertension due to hematological malignancies. This study included 11 patients with hematological malignancies whose portal hypertensions were alleviated and the following complications resolved via TIPS and portal vein stent placements. Both TIPS and portal vein stent placement can be effective in treating patients with portal hypertension complications caused by hematological diseases.
